# A putative new SARS-CoV protein, 3c, encoded in an ORF overlapping ORF3a

**DOI:** 10.1099/jgv.0.001469

**Published:** 2020-07-15

**Authors:** Andrew E. Firth

**Affiliations:** ^1^​ Division of Virology, Department of Pathology, Addenbrooke’s Hospital, University of Cambridge, Cambridge, UK

**Keywords:** SARS-CoV, coronavirus, overlapping gene, ORF3c, 3c, sarbecovirus

## Abstract

Identification of the full complement of genes in severe acute respiratory syndrome coronavirus 2 (SARS-CoV-2) is a crucial step towards gaining a fuller understanding of its molecular biology. However, short and/or overlapping genes can be difficult to detect using conventional computational approaches, whereas high-throughput experimental approaches – such as ribosome profiling – cannot distinguish translation of functional peptides from regulatory translation or translational noise. By studying regions showing enhanced conservation at synonymous sites in alignments of SARS-CoV-2 and related viruses (subgenus *Sarbecovirus*) and correlating the results with the conserved presence of an open reading frame (ORF) and a plausible translation mechanism, a putative new gene – ORF3c – was identified. ORF3c overlaps ORF3a in an alternative reading frame. A recently published ribosome profiling study confirmed that ORF3c is indeed translated during infection. ORF3c is conserved across the subgenus *Sarbecovirus*, and encodes a 40–41 amino acid predicted transmembrane protein.

## Findings

**Fig. 1.**
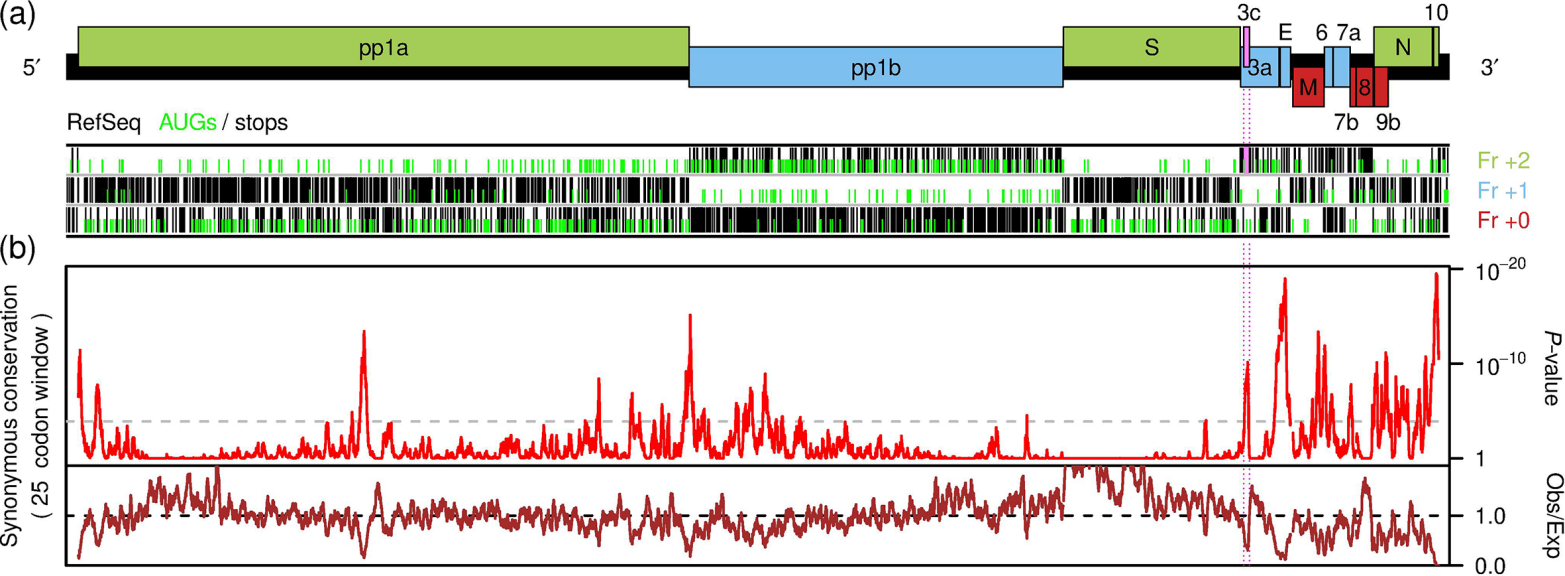
Synonymous site conservation analysis of sarbecoviruses. (a) Map of the SARS-CoV-2 genome (29 903 nt; black rectangle). Known ORFs are overlaid in red, blue or green depending on their relative reading frames (+0, +1, +2, respectively). Below are shown the positions of AUG (green) and stop (black) codons in each of the three reading frames, as indicated, in the reference sequence NC_045512.2. The putative ORF3c is indicated in pink (reading frame +2). (b) Synonymous site conservation analysis of 54 aligned sarbecovirus sequences. The red line shows the probability that the observed conservation could occur under a null model of neutral evolution at synonymous sites, whereas the brown line depicts the ratio of the observed number of substitutions to the number expected under the null model. The horizontal dashed grey line indicates a *P*=0.05 threshold after an approximate correction for multiple testing, namely scaling by (25 codon window size)/(length of plot in codons). Prior to analysis, the alignment was mapped to NC_045512.2 coordinates by removing alignment positions in which NC_045512.2 contained a gap character. NCBI accession numbers: NC_045512.2, AY274119.3, DQ022305.2, DQ071615.1, DQ084199.1, DQ084200.1, DQ412042.1, DQ412043.1, DQ648856.1, DQ648857.1, GQ153539.1, GQ153540.1, GQ153541.1, GQ153542.1, GQ153543.1, GQ153544.1, GQ153545.1, GQ153546.1, GQ153547.1, GQ153548.1, GU190215.1, JX993987.1, JX993988.1, KC881006.1, KF294457.1, KF367457.1, KF569996.1, KJ473811.1, KJ473812.1, KJ473813.1, KJ473816.1, KP886808.1, KP886809.1, KT444582.1, KY352407.1, KY417142.1, KY417143.1, KY417145.1, KY417146.1, KY417147.1, KY417148.1, KY417149.1, KY417150.1, KY417151.1, KY417152.1, KY770858.1, KY770859.1, KY770860.1, MG772933.1, MG772934.1, MK211374.1, MK211376.1, MK211377.1 and MK211378.1.

The aetiological agent of coronavirus disease 2019 (COVID-19) is the virus severe acute respiratory syndrome coronavirus 2 (SARS-CoV-2), a coronavirus in the genus *Betacoronavirus*, subgenus *Sarbecovirus*. Like other coronaviruses, SARS-CoV-2 has a positive-sense RNA genome that is approximately 30 000 nt in size. The 5′ two-thirds of the genome contain two long open reading frames (ORFs), ORF1a and ORF1b, which are translated from the viral genomic RNA (gRNA). ORFs 1a and 1b encode the polyproteins pp1a and pp1ab, where translation of pp1ab depends on a proportion of ribosomes making a programmed −1 nt ribosomal frameshift near the end of ORF1a to enter ORF1b. Polyproteins pp1a and pp1ab are proteolytically processed to produce the viral replication proteins (reviewed in [1]). The 3′ third of the genome contains a number of ORFs that encode the viral structural and accessory proteins. These ORFs are translated from a nested series of subgenomic mRNAs (sgmRNAs) that are produced during the infection cycle (reviewed in [2]). In SARS-CoV-2, these ORFs comprise S, 3a, E, M, 6, 7a, 7b, 8, N, 9b and possibly 10 (Fig. 1a). The S, E, M and N ORFs encode respectively the virus spike, envelope, membrane and nucleocapsid proteins – key components of the virus particle that are conserved among coronaviruses. ORFs 3a, 6, 7a, 7b, 8 and 9b encode accessory proteins, also present in the 2002–2003 severe acute respiratory syndrome coronavirus (hereafter SARS-CoV-1 to avoid any confusion), but less widely conserved across coronaviruses as a whole (reviewed in [3]). These 3′-proximal ORFs each have a corresponding dedicated sgmRNA, except for ORFs 7b, 9b and 10. ORFs 7b and 9b appear to be translated from the ORF7a and N sgmRNAs, respectively, via a leaky scanning mechanism [4, 5] whereas the translation mechanism for ORF10 is unknown.

There is considerable variability between coronavirus genera and subgenera in the complement of 3′-encoded accessory genes [3]. Even within the sarbecovirus subgenus, there are differences. For example, SARS-CoV-1 has an ORF3b that overlaps the 3′ region of ORF3a but is truncated or absent in SARS-CoV-2. Also, in many human-adapted SARS-CoV-1 isolates, ORF8 is split by a frame-disrupting deletion [6]. ORF10 is apparently translated in SARS-CoV-2 [7] but is truncated in SARS-CoV-1. Identification of the full complement of genes in SARS-CoV-2 is a crucial step towards gaining a fuller understanding of its molecular biology, and may also help guide vaccine or other antiviral strategies. This information also facilitates rational manipulation of the viral genome (e.g. for developing replicon systems or for mutagenesis studies). However, short and/or overlapping genes can be particularly difficult to identify using traditional computational approaches. On the other hand, high-throughput experimental techniques such as ribosome profiling and high resolution mass spectrometry – while powerful – do not necessarily distinguish between functional proteins, regulatory translation (where it is the act of translation rather than the encoded product that is biologically relevant) and translational noise.

Comparative genomics offers a way forward: analysis of patterns of substitutions across alignments of related sequences can be used to reveal the signatures of ‘hidden’ protein-coding genes. Analysis of synonymous substitution rates provides a particularly sensitive technique for identifying *overlapping* functional elements embedded within protein-coding genes, because such elements constrain synonymous changes that are otherwise selectively more-or-less neutral [8]. When combined with the conserved presence of an ORF and the conserved presence of a plausible translation mechanism, overlapping genes may be distinguishable from overlapping non-coding elements such as functionally important RNA structures [9–12].

Sequences with 100 % coverage of, and ≥70 % amino acid identity to, the SARS-CoV-2 pp1ab sequence were identified with NCBI tblastn [13] on 12 January 2020 and downloaded. These cut-off thresholds corresponded precisely to the subgenus *Sarbecovirus*. Sequences that did not cover the entire protein-coding region of the genome, and all sequences with >99 % amino acid identity in pp1ab to SARS-CoV-1 except a single reference sequence NC_004718.3 were removed. This left 54 sequences (SARS-CoV-1, SARS-CoV-2 and 52 bat coronaviruses). Codon-respecting alignments were produced using a previously described procedure [8]. In brief, each individual genome sequence was aligned to the SARS-CoV-2 reference sequence (GenBank accession number NC_045512.2) using code2aln version 1.2 [14]. Genomes were then mapped to NC_045512.2 coordinates by removing alignment positions that contained a gap character in the reference sequence, and these pairwise alignments were combined to give the multiple sequence alignment. To assess conservation at synonymous sites, the known virus coding regions were extracted from the alignment (with codons selected from the longer ORF in each overlap region) and concatenated in-frame, and the alignment was analysed with synplot2 [8] using a 25 codon sliding window. Conservation statistics were then mapped back to NC_045512.2 coordinates for plotting (Fig. 1b).

The synonymous site conservation analysis revealed conserved features towards the 5′ end of ORF1a, in the middle of ORF1a, near the end of ORF1a and start of ORF1b, and in many parts of the 3′ region of the viral genome. Most of these conserved elements do not correspond to conserved overlapping ORFs and likely represent functional RNA sequence elements – a case in point being the frameshift-stimulating RNA pseudoknot [15] at the junction of ORFs 1a and 1b. However, a peak in conservation within ORF3a stood out because, although short, it coincides with an overlapping alternative-frame AUG-initiated ORF – hereafter ORF3c – positioned close to the 5′ end of ORF3a, where it might be accessible via ribosomal leaky scanning (reviewed in [16]).

**Fig. 2.**
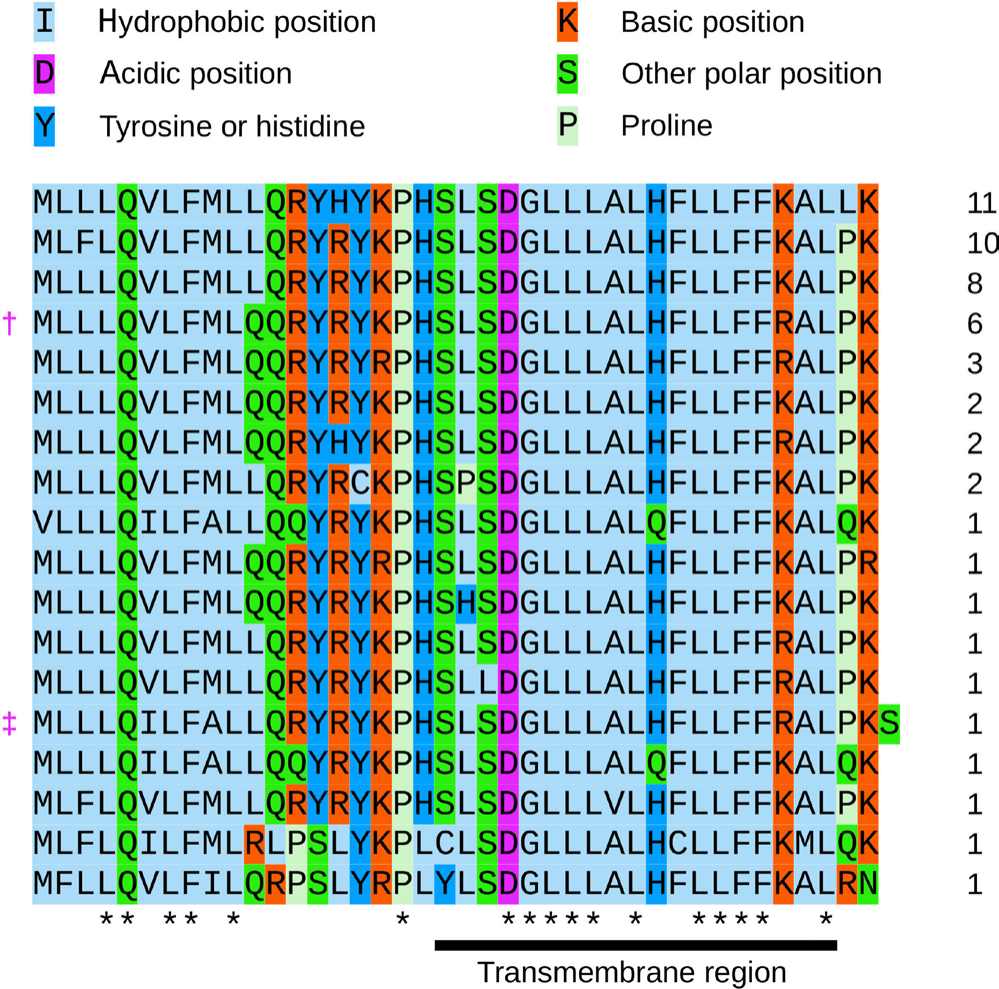
Amino acid alignment of sarbecovirus 3c sequences. Amino acids are colour-coded according to their physicochemical properties. Asterisks indicate completely conserved columns in the alignment. The transmembrane region predicted by Phobius is indicated with a black bar below the alignment. Numbers at the right indicate the number of times the particular sequence occurs among the 54 sarbecovirus sequences (see Fig. 1 caption for accession numbers). †, SARS-CoV-1. ‡, SARS-CoV-2. For the sequence beginning with GUG instead of AUG, the genetic decoding (i.e. valine) is shown, even though non-AUG initiation codons are normally expected to be decoded as methionine by initiator Met-tRNA.

Closer inspection revealed that the presence and location of the 3c initiation and stop codons were conserved across sarbecoviruses. The 3c AUG codon is present in all but 1 of the 54 sequences, where it is replaced with GUG (MG772933, a bat coronavirus). In all 54 sequences, there is an A at the −3 position, giving a strong initiation context [17]. The GUG in MG772933 may also serve as an initiation site, as GUG codons can be utilized for initiation – at a reduced efficiency compared to AUG – when in a strong initiation context (reviewed in [16]). In 52 sequences there are 2 upstream AUGs – the ORF3a AUG (intermediate context; C or U at −3 and G at +4) and another AUG also in the ORF3a reading frame (weak context; U at −3 and A at +4); in the remaining two sequences the second of these AUGs is absent. The lack of a strong initiation context for either of the upstream AUG codons might enable a proportion of preinitiation scanning 43S complexes loaded on the ORF3a sgmRNA to leaky scan to the 3c initiation codon. The 3c ORF has a length of 40 codons in all 54 sequences except 1, namely SARS-CoV-2, where it is 41 codons in length (Fig. 2). The SARS-CoV-2 putative 3c protein has a molecular mass of 4.9 kDa and a pI of 10.9. The protein is also predicted to contain a transmembrane domain (via Phobius [18]) (Fig. 2). Curiously, the transmembrane amino acids are relatively highly conserved, suggesting that they may form interactions within the lipid bilayer, for example, membrane-disrupting or membrane-associated signalling activities.

**Fig. 3.**
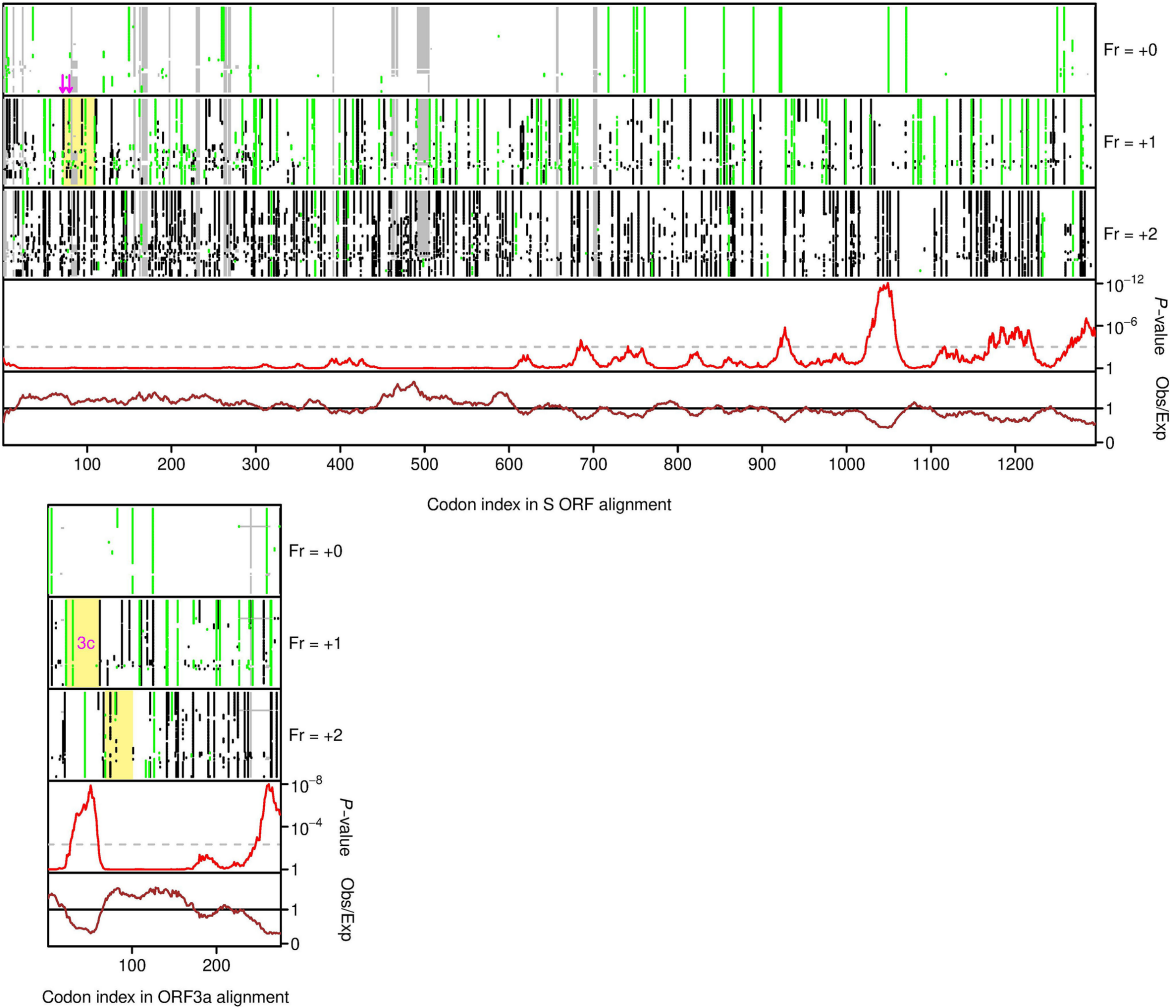
Conservation analyses of the sarbecovirus S and 3a ORFs. In each plot, the upper three panels show the positions of alignment gaps (grey), stop codons (black) and AUG codons (green) in each reading frame in each of the 54 aligned sequences. In these plots the canonical ORFs (i.e. S and 3a) are taken as reading frame +0. Below is shown the analysis of conservation at synonymous sites (see Fig. 1 caption for details). In contrast to Fig. 1, here all alignment gaps were retained instead of mapping to NC_045512.2 coordinates. Novel alternative-frame translated ORFs identified in the SARS-CoV-2 ribosome profiling study of Finkel *et al*. [7] are indicated with yellow rectangles; for S, the two in-frame alternative initiation sites are indicated with pink arrows. ORF3c is labelled. Note that only ORF3c has conserved start and stop codon positions across sarbecoviruses and only ORF3c coincides with a region of enhanced synonymous site conservation.

Ribosome profiling is a high-throughput sequencing technique that allows footprinting of initiating and/or elongating ribosomes at sub-codon resolution and hence the global identification of initiation sites and/or sequence regions and reading frames undergoing translation [19, 20]. A recent ribosome profiling study of cells infected with SARS-CoV-2 revealed 23 novel translated ORFs [7]. Ten are very short (≤15 codons). Seven of the remainder comprise 5′ extensions or 5′ truncations of previously known ORFs (M, 6, 7a, 7b, 9b and 10). Two are uORFs positioned on the gRNA upstream of ORF1a that may play a role in regulating ORF1a/1b expression as previously proposed for uORFs in other coronaviruses [19, 21]. After excluding these ORFs, only four novel translated ORFs remain: ORF3c (25 457–25 582; 41 codons), another ORF overlapping ORF3a (25 596–25 697; 33 codons), an ORF overlapping the S ORF (21 74421 863; 39 codons) and a truncated version of the same (21 768–21 863; 31 codons) (where numbers indicate ORF coordinates in NC_045512.2). To investigate the four overlapping ORFs, the ORF S and ORF 3a regions were extracted from all 54 sarbecovirus sequences, translated to amino acids and aligned using muscle [22], and the amino acid alignments were used to guide codon-respecting nucleotide sequence alignments (EMBOSS tranalign [23]). These alignments were analysed with synplot2, again using a 25 codon window size. Of the four translated novel ORFs (Fig. 3, yellow rectangles), only ORF3c was found to coincide with a synonymous site conservation signal. Moreover, the other three novel ORFs are not conserved: in many sarbecovirus sequences they lack the AUG codon and are interrupted by stop codons (Fig. 3).

Although synonymous site conservation can result from overlapping non-coding or coding elements, the conserved presence and conserved positions of the ORF3c start and stop codons suggests the latter interpretation. Moreover, the ribosome profiling study of Finkel *et al*. [7] confirms that ORF3c is indeed translated during infection. The combination of comparative genomics showing purifying selection (which to a large extent is synonymous with functional importance) and ribosome profiling showing expression strongly suggests that 3c is a functional protein, conserved throughout sarbecoviruses. While the known SARS-CoV-2 genes have already been investigated in SARS-CoV-1 (reviewed in [3]), 3c has never before been studied. Clearly, additional work with SARS-CoV reverse genetics systems will be required to elucidate the 3c protein function, and it may eventually provide a new target for vaccine or antiviral strategies. The synplot2 analysis (Fig. 1b) has also revealed other functional elements embedded within the viral protein-coding genes (e.g. in ORF1a), which may also be worthy of experimental investigation.

During preparation of this manuscript, 3c was independently discovered by Cagliani *et al*. [24] (where it is termed 3h), who performed a similar analysis with synplot2 but used far fewer sarbecovirus sequences, and hence achieved lower statistical significance for conserved elements. More recently, 3c was also independently discovered by Jungreis *et al*. [25] (where it is also termed 3c) using PhyloCSF, in which ORF3c–frame codon substitutions are compared with coding and non-coding evolutionary models, thus representing an independent approach that is completely different from that used in synplot2.
